# Oxidative Stress and Inflammation in Obesity, Diabetes, Hypertension, and Related Cardiometabolic Complications

**DOI:** 10.1155/2014/506948

**Published:** 2014-02-27

**Authors:** Joseph Fomusi Ndisang, Alfredo Vannacci, Sharad Rastogi

**Affiliations:** ^1^Department of Physiology, University of Saskatchewan College of Medicine, 107 Wiggins Road, Saskatoon, SK, Canada S7N 5E5; ^2^Department of Pharmacology, Center for Integrative Medicine, Center for Molecular Medicine (CIMMBA), University of Florence, Viale Pieraccini 6, 50139 Florence, Italy; ^3^Division of Cardiology, Department of Medicine, Henry Ford Heart and Vascular Institute, 2799 West Grand Boulevard, Detroit, MI 48202-2689, USA

Many chronic diseases are characterized by excessive oxidative stress and inflammation [1–3]. The recent escalation of chronic conditions like obesity, insulin resistance, diabetes, hypertension, and other related cardiometabolic complications in all ages of the population including children, adolescences, and adults poses a great challenge to health care systems [4, 5]. Many cardiometabolic complications are multifactorial diseases and a wide variety of etiological factors including genetic, habitual, environmental, and epigenetic may be involved [4, 5]. Although these factors perturb the physiological milieu in different ways, it has been consistently shown that a common denominator amongst these factors is the production of increased oxidative stress and inflammation at various stages during the progression and development of many cardiometabolic disorders [4–8]. Although significant strides have been made in elucidating the role of oxidative stress and inflammation in insulin resistance, diabetes, and many cardiometabolic diseases, novel mechanistic studies are needed to broaden our knowledge on the treatment and management of these chronic conditions.

This special issue contains review papers and research articles that address a broad range of mechanistic paradigms in the pathophysiology of insulin resistance, diabetes, hypertension, obesity, and related cardiometabolic complications. The role of inflammation and apolipoproteins in the development of atherosclerotic plague, thrombosis, and related cardiac complications has been widely acknowledged [9–11].

Since apolipoproteins play a major pathophysiological role in atherosclerosis, in an article featuring in this special issue, A. J. Lepedda et al. used two-dimensional electrophoresis (2DE) coupled with Matrix-Assisted Laser Desorption/Ionization (MALDI), Time of Flight (TOF), and Mass Spectrometry (MS) analysis to characterize the apolipoprotein components of very-low-density lipoprotein (VLDL), low-density lipoprotein (LDL), and high-density lipoprotein (HDL) from plasma of patients undergoing carotid endarterectomy. The study of A. J. Lepedda et al. will unveil novel perspectives on the pathophysiological role of apolipoproteins in development of atherosclerotic plague. In another related article featuring in this special issue, W. Sun et al. showed that a herbal extract obtained from the medicinal plant *Magnolia officinalis *attenuated cardiac hypertrophy and cardiac dysfunction by reducing myocardial lipid accumulation, inflammation, oxidative stress, and apoptosis in animals fed with high-fat diet. Interestingly, the benefit of *Magnolia officinalis* is not limited to myocardial dysfunction. In another study by W. Cui et al. reported in this special issue, *Magnolia officinalis* was shown to abate several markers of inflammation and oxidative stress in the kidneys including tumor necrosis factor-*α*, plasminogen activator inhibitor-1, 3-nitrotyrosine, and 4-hydroxy-2-nonenal. Interestingly, the suppression of these prooxidative/inflammatory agents were associated with the potentiation of peroxisome proliferator-activated receptor-*γ* coactivator-1*α* and hexokinase II, with improved renal morphology and the reduction of proteinuria suggesting improved renal function.

An important cytoprotective enzyme with benefits against diabetes, hypertension, obesity, and related cardiometabolic complications is heme oxygenase [1–3, 12–15]. In a related article featuring in this special issue, S. Tiwari et al. showed that the heme oxygenase system improves cardiac function by attenuating markers of heart failure, cardiac hypertrophy/lesions, extracellular matrix/profibrotic proteins, and inflammatory/oxidative mediators, while concomitantly enhancing adiponectin and atrial natriuretic peptide in obese rats with insulin resistance. Similarly, Y. Son et al. wrote a review article that delineates the protective mechanisms of heme oxygenase in metabolic diseases. Furthermore, Md. J. Uddin and coworkers demonstrated in a research article that carbon monoxide, a product generated by heme oxygenase, suppressed inflammation in colitis mice model via inhibition of glycogen synthase kinase-3, a serine-threonine protein kinase implicated in glycogen metabolism, and other cellular functions including apoptosis [16, 17]. Besides the heme oxygenase system, other molecules including trace elements are important in the regulation of cardiometabolic functions [18, 19]. Emerging evidence indicates that perturbations in the delicate balance of trace elements such as copper and zinc may offset redox equilibrium in physiological milieu leading to oxidative stress and eventually to the development of diabetes and diabetic complications [20–22]. In this special issue, J. Xu et al. reported novel insights on zinc/copper ratio in patients with different pathological profiles including impaired fasting glucose, impaired glucose tolerance type-1 diabetes, and type-2 diabetes.

Collectively, the articles presented in this special issue would unveil novel concepts that would stimulate further research in this area of considerable interest given the escalation of diabetes, obesity, hypertension, and related cardiometabolic complications in all ages of the population including children.

*Joseph Fomusi Ndisang* *Joseph Fomusi Ndisang* *Alfredo Vannacci* *Alfredo Vannacci* *Sharad Rastogi* *Sharad Rastogi*
